# A Radial Zoom Motion-Based Paradigm for Steady State Motion Visual Evoked Potentials

**DOI:** 10.3389/fnhum.2019.00127

**Published:** 2019-04-16

**Authors:** Xiaoke Chai, Zhimin Zhang, Kai Guan, Guitong Liu, Haijun Niu

**Affiliations:** ^1^School of Biological Science and Medical Engineering, Beihang University, Beijing, China; ^2^Beijing Advanced Innovation Center for Biomedical Engineering, Beihang University, Beijing, China; ^3^State Key Laboratory of Virtual Reality Technology and Systems, Beihang University, Beijing, China

**Keywords:** brain-computer interface, electroencephalogram, steady-state visual evoked potential, steady-state motion visual evoked potential, fatigue

## Abstract

**Background:** In steady state visual evoked potential (SSVEP)-based brain-computer interfaces, prolonged repeated flicker stimulation would reduce the system performance. To reduce the visual discomfort and fatigue, while ensuring recognition accuracy, and information transmission rate (ITR), a novel motion paradigm based on the steady-state motion visual evoked potentials (SSMVEPs) is proposed.

**Methods:** The novel SSMVEP paradigm of the radial zoom motion was realized using the sinusoidal form to modulate the size of the stimuli. The radial zoom motion-based SSMVEP paradigm was compared with the flicker-based SSVEP paradigm and the SSMVEP paradigm based on Newton's ring motion. The canonical correlation analysis was used to identify the frequency of the eight targets, the recognition accuracy of different paradigms with different stimulation frequencies, and the ITR under different stimulation durations were calculated. The subjective comfort scores and fatigue scores, and decrease in the accuracy due to fatigue was evaluated.

**Results:** The average recognition accuracy of the novel radial zoom motion-based SSMVEP paradigm was 93.4%, and its ITR reached 42.5 bit/min, which was greater than the average recognition accuracy of the SSMVEP paradigm based on Newton's ring motion. The comfort score of the novel paradigm was greater than both the flicker-based SSVEP paradigm and SSMVEP paradigm based on Newton's ring motion. The decrease in the recognition accuracy due to fatigue was less than that of the SSSMVEP paradigm based on Newton's ring motion.

**Conclusion:** The SSMVEP paradigm based on radial zoom motion has high recognition accuracy and ITR with low visual discomfort and fatigue scores. The method has potential advantages in overcoming the performance decline caused by fatigue.

## Introduction

The brain-computer interface (BCI) allows for direct communication between the brain and external devices. In particular, recent BCIs based on electroencephalogram (EEG) signals have high application value in neuro engineering and rehabilitation (Daly and Huggins, 2015). The paradigm design of the BCI is the key issues to the research and development of BCI systems. The commonly used reactive BCIs are realized though specific audio or visual tasks, inducing corresponding event-related potentials (ERPs) in the brain (Celesia et al., 1996), which can be identified to determine the communication or control intention (Muller and Hillyard, 2000). Visual stimuli can induce electrical responses in the occipital cortex, called visual evoked potentials (VEPs). A single visual stimulus can induce a transient visual evoked potential (TVEP), while repeated visual stimuli (RVS) at a certain frequency can be used to induce a steady state visual evoked potential (SSVEP) (Zhu et al., 2010). The paradigm based on VEPs has been widely used as it is a natural response of the visual pathway without any training. The RVS in the SSVEP paradigm is designed using color alternation or graphic flicker at a certain frequency (Celesia et al., 1996). Its periodic spectrum characteristics are not easily affected by eye blinks or other artifacts, and thus can be used to achieve high recognition accuracies and information transmission rates (ITR) (Cheng et al., 2002; Vialatte et al., 2010; Ng et al., 2012; Sengelmann et al., 2017).

However, as BCI is a human-computer interaction system, the comfort interface of the SSVEP paradigm is also important. During frequent use of the SSVEP interaction paradigm for a long time, the subject passively experiences repeated flicker stimulation, which reduces the performance due to visual fatigue and eye discomfort (Punsawad and Wongsawat, 2012). In order to alleviate the visual discomfort caused by flicker-based SSVEP paradigm, Xie et al. proposed a BCI paradigm based on steady state motion visual evoked potentials (SSMVEP) (Xie et al., 2012). Similar to the perception of light and contrast, the visual system is also sensitive to the perception of motion (Kremláek et al., 2004). Heinrich et al. demonstrated that SSMVEP can quickly record the brain's response to motion without obvious adaptation phenomena, which can significantly reduce visual fatigue (Heinrich and Bach, 2003). Using periodic Newtonian ring motion as an SSMVEP paradigm (Xie et al., 2012), as the lower stimulation intensity may alleviate visual fatigue and discomfort, a control accuracy of 86% for a four target control system can be obtained. Compared with the flicker-based SSVEP paradigm (Xie et al., 2016), the results showed that although the SSMVEP-BCI has a lower accuracy than that of the SSVEP-BCI system, the amplitude decrease due to fatigue is more significant in SSVEP compared with SSMVEP. However, the comparison did not consider the shape of the stimuli and as previous studies have showed, stimuli with contours and sharp edges have a more sensitive visual response (Regan, 2000), and the SSVEPs of circular and square stimulations are different (Bieger and Molina, 2010). The different performances of circular-based SSMVEP and square-based SSVEP paradigms may be related to the shape of the stimulus pattern, and an SSMVEP based on various graphical shapes should be compared.

Moreover, the SSMVEP induced by different motor paradigm is related to the motion onset visual evoked potentials (mVEP) at the start time of the motion, which reflect the brain's processing mechanism of motion information (Snowden and Freeman, 2004). Beveridge et al. demonstrated that the start time of horizontal or radial motions can induce mVEPs (Beveridge et al., 2016). Among these, the expansion and contraction motions are the typical radial motions that are experienced as the relative distance changes along the visual axis, and can produce the greatest response. Yan et al. proposed several SSMVEP paradigms based on a circular swing, a spiral, and rotation (Yan et al., 2018), and showed that any stimulus with periodic motion can induce SSMVEP, while the recognition accuracy of the paradigm based on radial contraction-expansion was 80.7%. The low recognition accuracy of the SSMVEP based on radial motion may be related to the graphic itself, and whether these complex graphical motion paradigms are superior to the flicker-based paradigm for visual comfort. There are no studies on SSMVEPs based on simple graphical radial motion. Methods to reduce visual discomfort and fatigue, while ensuring recognition accuracy and ITR, are yet to be realized (Chang et al., 2014).

In the present study, a paradigm that uses the sinusoidal form to modulate the block stimulus size to achieve periodic radial zoom motion is proposed. The performance of the novel SSMVEP stimulation paradigm based on simple graphical radial zoom motion was compared to the flicker-based SSVEP paradigm and the SSMVEP paradigm based on Newton's ring motion. Due to the difference caused by the shape, each kind of paradigm has two stimulation shapes; square and circular blocks. The recognition accuracies, subjective comfort scores, and decreases in the recognition accuracy due to fatigue were evaluated for all of the paradigms.

## Method

### Paradigm Design

The psych toolbox in MATLAB (Mathworks Inc.) was used to design the six stimulus paradigms (Brainard, 1997), in which the motion modes were the square block flicker, square block radial zoom motion, square Newton's ring motion and circle block flicker, circle block radial zoom motion, and Newton's ring motion.

The flicker stimulus sequence was generated by modulating the luminance of the stimulus block (Chen et al., 2014), and the radial zoom motion was generated by modulating the size of the stimulus block. For the flicker, the dynamic range of the stimulation luminance was 0–1, where 0 and 1 represent darkness and the highest luminance, respectively. For the zoom motion, the dynamic range of the stimulation size was from 0 to 1, where 0 and 1 represent the minimum and maximum sizes, respectively. We define the stimulus frequency, *f*; screen refresh rate, *r*; current frame number, *i*; and stimulation signal, ∅ (*i*) was calculated as in equation (1):

(1) $$\begin{array}{l} \emptyset \left(i\right)=abs\left(\sin\left(\pi *\frac{f}{2}*\left(\frac{i}{r}\right)\right)\right)\;\left(i=1,2,\ldots ,60\right) \end{array}$$

The square ring stimulus was generated using the modulation method of Newton's ring motion (Xie et al., 2012). The square ring stimulus is made up of a series of concentric black and white squares, and is given by equation (2):

(2) $$\begin{array}{l} E=I_{\max}[(sin(cos{\left(2*\pi *\frac{d}{\text{λ}}+\emptyset (i)*\frac{\pi }{2}\right)}^{2}-0.5)] \end{array}$$

where λ is a constant equal to 0.05, and *d* is a 360 × 360 square matrix. The ring oscillation motion is formed by ∅(*i*). When ∅(*i*) increases with *i*, its phase shifts from 0 to π, and Newton's ring motion is achieved with a phase shift from π back to 0.

**Figure 1:**
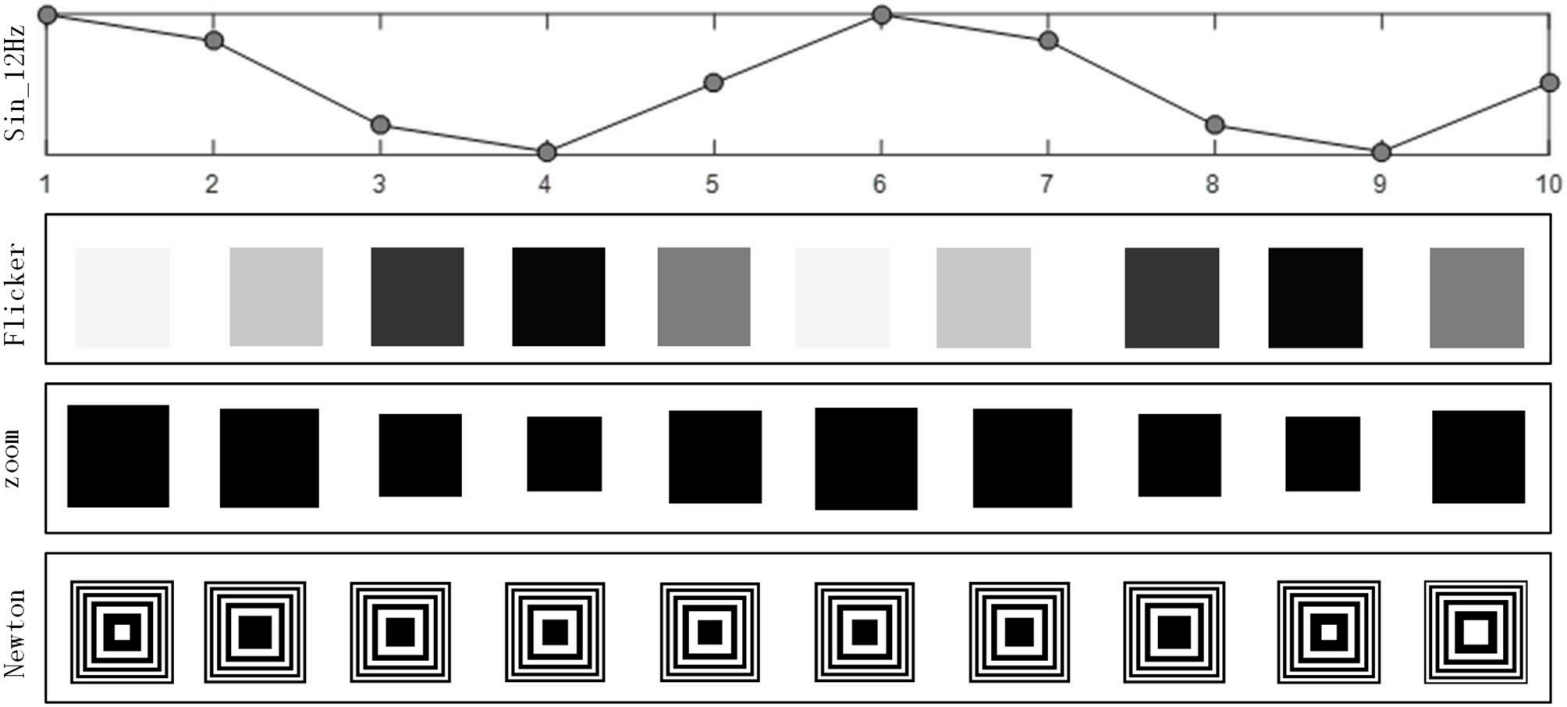
Each frame of the 12 Hz stimulation for different paradigms.

The stimuli reversal procedure in one stimulus period is shown in Figure 1, as for a 12 Hz motion reversal frequency there are ten frames per cycle. The first line shows the flicker-based SSVEP paradigm; the size of the block for each of the ten frames was the same, while the luminance changed with the sinusoidal function modulation. The second line shows the SSMVEP paradigm based on radial zoom motion; the block luminance in each of the ten frames was the same (a white block in a black background), while the side length changed corresponding to the sinusoidal function modulation. The third line shows the SSMVEP paradigm based on Newton's ring motion, with the square ring changing.

### Experiment Protocol

Four males and four females (22–27 years old) were recruited as subjects in this study. The participants were right-handed, healthy, and had normal color and visual perception. No subjects had previously participated in any SSVEP-based BCI experiments. This study was carried out in accordance with the recommendations of Beihang University Ethics Committee with written informed consent from all subjects. All subjects gave written informed consent in accordance with the Declaration of Helsinki. The protocol was approved by Beihang University Ethics Committee.

The EEG signals were sampled at 1000 Hz using Neuroscan (USA). During the experiment, the electrode impedances were kept below 10 kΩ before data recording. EEGs were collected from the following eight channels: PO8, PO4, PO7, PO8, POz, O1, Oz, and O2. The experiment was performed in a quiet room, the stimulus paradigm were presented on a LCD screen (23.6 inch, 1,080 pixels) with a refresh rate of 60 Hz, and the subjects were positioned 70 cm from the screen.

**Figure 2:**
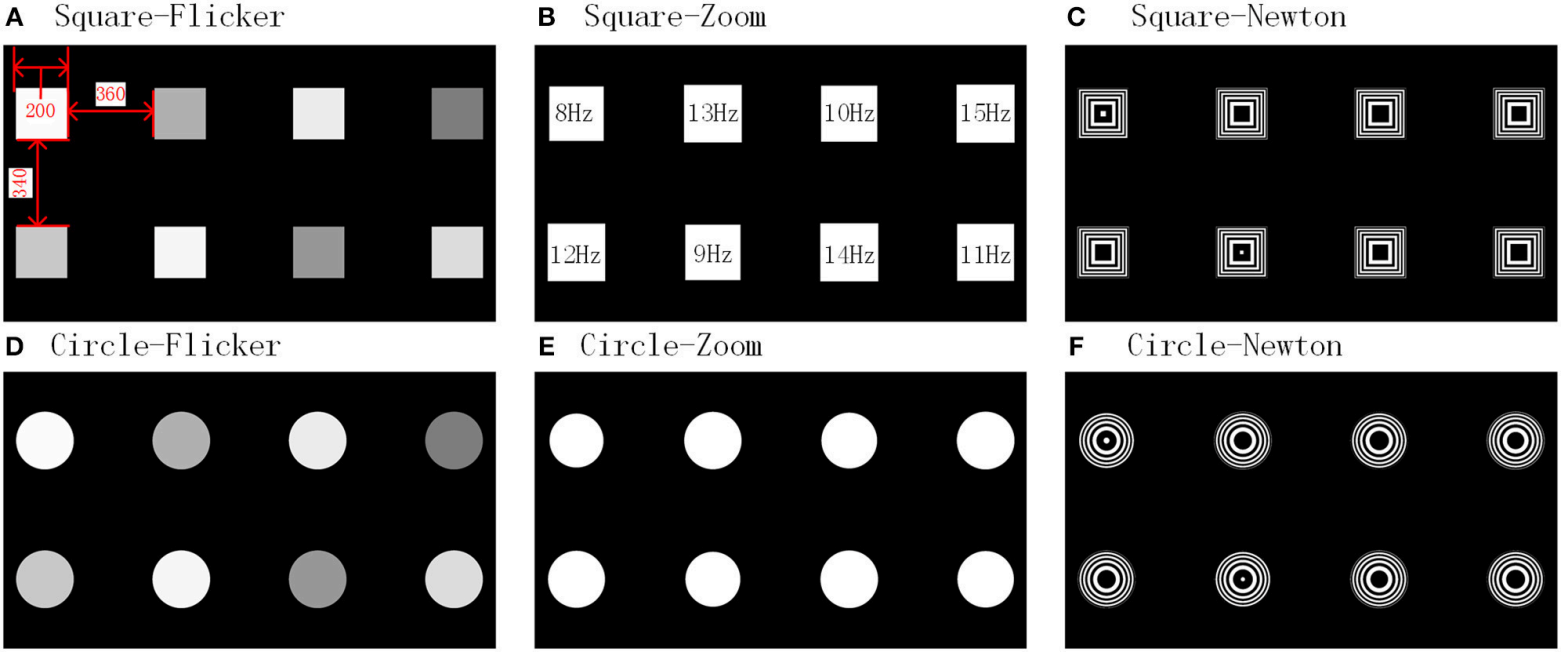
Paradigm interfaces.

**Figure 3:**
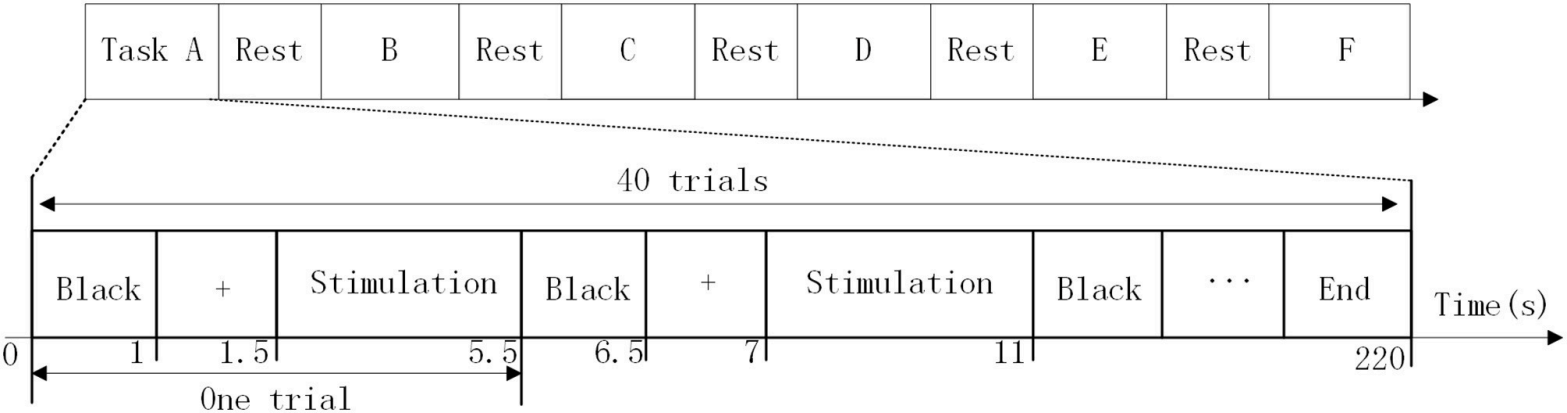
Experimental time sequence.

Each paradigm set was comprised of eight stimulus targets (with a flip frequency of 8–15Hz and frequency interval of 1 Hz). The size and location of the stimulus block are shown in Figure 2. The Experimental time sequence are shown in Figure 3. Subjects were asked to stare at one target for 4 s per trial, with an interval of 1.5 s, and then stare at another stimuli. For each paradigm run, there were 40 trials with five trials for each target. Before the start of each trial, a red “+” symbol appeared randomly at the location of the stimulus target for a duration of 0.5 s, then the block of the corresponding position was presented for 4 s as a single trial. Each trial was isolated by a black screen and the interval time was fixed to 1 s. Each paradigm set was isolated by rest and the interval time was 10 min. After finishing each paradigm set, the subject was asked to fill out a comfort questionnaire to obtain their feelings on the stimulation. They each provided a score between 1 (not) and 7 (very) for each of the following four questions after gazing at each of the stimulation in turn (Bieger and Molina, 2010):

- How much do you like this stimulation?
- How much will this stimulation increase your tiredness?
- How long could you look at this stimulation?
- How annoying is this stimulation?

### Data Processing

Band-passed filter between 3 and 40 Hz was used to preprocess the collected EEG. The average time-domain waveform of the SSMVEP was obtained by averaging all of the data segments for each stimulation frequency. The Welch power spectrum was then calculated using the average waveform of the SSVEP or SSMVEP.

To investigate the applicability of the proposed SSMVEP-based BCI, a canonical correlation analysis (CCA) was used for offline target detection (Lin et al., 2006). CCA is a non-parametric multivariable method to reveal the underlying correlation between two sets of multidimensional variables. In the study, EEG signals from eight occipital region channels; PO3, PO4, PO7, PO8, POz, O1, Oz, and O2, were calculated as one set of variables, presented at frequencies (*f*_*n*_) of 8, 9, 10, 11, 12, 13, 14, and 15 Hz, respectively. The reference signals (*Yf*_*n*_) were composed of sinusoid and cosinusoid pairs at the frequency of the stimulus and its second harmonics, as in Equation (3):

(3) $$\begin{array}{r} Y_{n,\;h\;}=\left\{\;\begin{array}{c} \sin\left(2\pi \cdot f_{n}\cdot t\right)\\ \cos\left(2\pi {\cdot f}_{n}\cdot t\right)\\ \ldots \\ \ldots \\ sin\left(2\pi {\cdot hf}_{n}\cdot t\right)\\ cos\left(2\pi {\cdot hf}_{n}\cdot t\right) \end{array}\right\},\;t=\frac{1}{f_{s}} \end{array}$$

Where fn is the stimulus frequency, fs is the sample rate, n is the number of the target, and h is the number of harmonics. For SSVEP recognition, hϵ[1, 2] while for SSMVEP recognition hϵ[0.5, 1].

The information transfer rate (ITR) [34] was calculated to evaluate the BCI system, and is given by Equation (4):

(4) $$\begin{array}{r} \text{ITR}=\frac{60}{T}\left[{\text{log}}_{2}N+P{\text{log}}_{2}P+\left(1-P\right){\text{log}}_{2}\left(\frac{1-P}{N-1}\right)\right] \end{array}$$

Where *P* is the recognition accuracy, *N* is the number of targets, and *T* is the time. In this study *N* = 8 and the ITRs under different durations (*t* = 0.5, 1, 1.5, 2, 2.5, 3, 3.5, and 4 s) were calculated.

### Statistical Analysis

The average recognition accuracy of the SSVEP or SSMVEP from the 1–10, 11–20, 21–30, and 31–40 trials of each run, representing fatigue levels 1, 2, 3, and 4, respectively, were calculated for all six paradigm tasks. The visual comfort scores of different paradigms were normalized. The average score of the visual comfort was calculated as the sum of the average scores of the two positive questions, A and C, and the two inverse questions, B and D:

(5) $$\begin{array}{r} \text{Comfort Score}=\left[A+\left(8-\text{B}\right)+C+\left(8-\text{D}\right)\right]/2.8 \end{array}$$

The fatigue score was calculated as the averages from questions B and C, given by:

(6) $$\begin{array}{r} \text{Fatigue Score}=\left[\text{B}+\left(8-\text{C}\right)\right]/1.4 \end{array}$$

The comfort score and average recognition accuracy, the fatigue score and decrease in the recognition accuracy (from fatigue levels 1 to 4) were analyzed. A paired *t*-test was performed for the recognition accuracy and comfort scores for all six paradigms. The difference between the two different shapes in the same stimulation paradigm was compared, and the difference among the three different paradigms in the same shape was compared. The confidence level was set to 95% and a *p* < 0.05 was considered to represent a statistic difference.

## Results

### Spectrum of the Steady-State Motion Visual Evoked Potential

**Figure 4:**
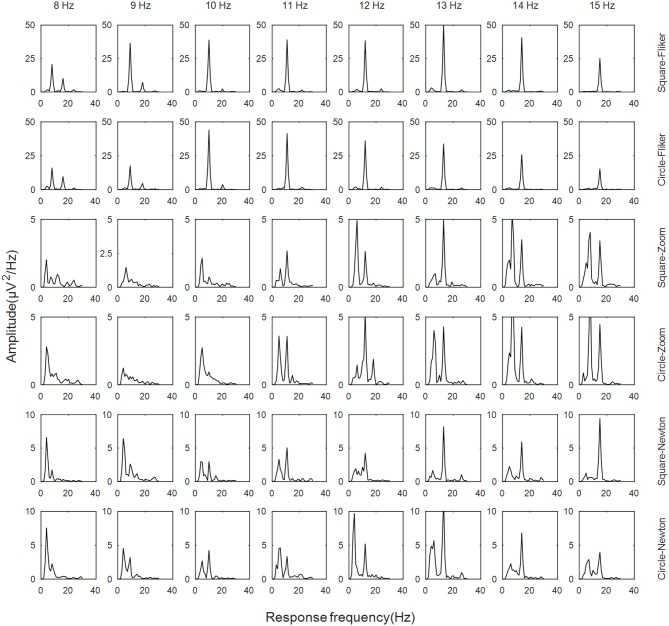
Spectrum of the six paradigms.

The frequency domain for the SSVEPs or SSMVEPs induced by the six paradigms was analyzed. Figure 4 shows the spectra of the different frequencies for subject 6 in channel Oz. It can be seen from the power spectrum that the amplitudes were significant at each frequency for the six paradigms, and there were also some harmonic components. The peak amplitude value of SSMVEP was smaller than the amplitude of the SSVEP induced by the square and circular flickers. For SSVEP there was significant amplitude at second harmonic, while the half harmonic component induced by SSMVEP was significant.

### Recognition Accuracy of Different Paradigms

The recognition accuracy of the eight subjects in different paradigms are shown in Table 1. The average recognition accuracy of the SSVEP paradigm based on flicker was 99.1%, and there were no significant differences between the circular and square stimuli. The average recognition accuracy of SSMVEP based on Newton's ring motion is 77.5%, and the average recognition accuracy of the square ring Newtonian motion was 76.3%. The average recognition accuracy of the SSMVEP based on the radial zoom motion of the square and circular stimuli were 89.7 and 93.4%, respectively, and there were also no significant differences between the different shapes. However, the recognition accuracy of the SSMVEP paradigm based on radial zoom motion was significantly greater than the SSMVEP paradigm based on Newtonian motion.

**Table 1:** Recognition accuracy of the different paradigms.

	**S1**	**S2**	**S3**	**S4**	**S5**	**S6**	**S7**	**S8**	**Average (%)**
SSVEP-square-flicker	100.0	95.0	100.0	97.5	100.0	100.0	100.0	100.0	99.1 ± 1.9
SSVEP-circle-flicker	100.0	95.0	100.0	100.0	97.5	100.0	100.0	100.0	99.1 ± 1.9
SSMVEP-square-zoom	97.5	95.0	97.5	82.5	82.5	90.0	97.5	75.0	89.7 ± 8.7
SSMVEP-circle-zoom	95.0	92.5	100.0	95.0	85.0	97.5	100.0	82.5	93.4 ± 6.5
SSMVEP-square-newton	77.5	65.0	90.0	72.5	70.0	90.0	95.0	50.0	76.3 ± 15.1
SSMVEP-circle-newton	75.0	70.0	85.0	82.5	77.5	92.5	92.5	45.0	77.5 ± 15.4

**Figure 5:**
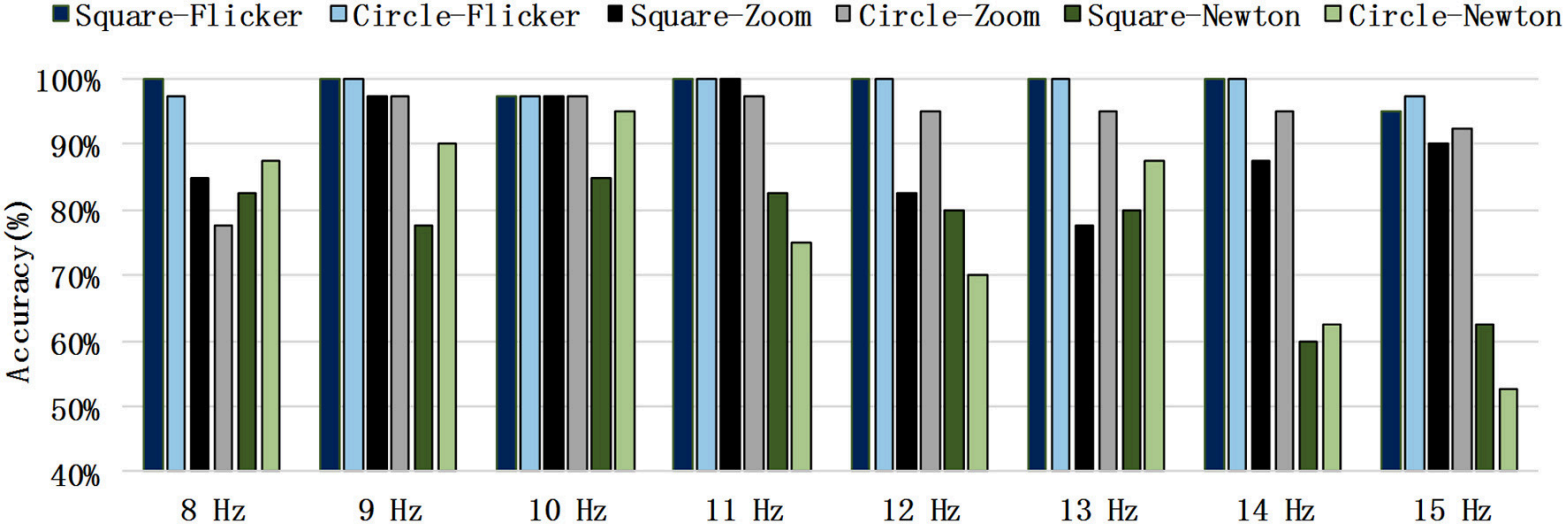
Recognition accuracy in each stimulus frequency.

The recognition accuracy of each paradigm for each stimulus frequency are shown in Figure 5. The frequency recognition accuracy of the SSMVEP based on the circular radial zoom motion was greater than that of the square motion. The recognition accuracy for almost all of the frequencies of the circular radial zoom motion were significantly greater than that of the SSMVEP paradigm based on Newton's ring motion.

### Information Transmission Rate for Different Stimulation Durations

**Figure 6:**
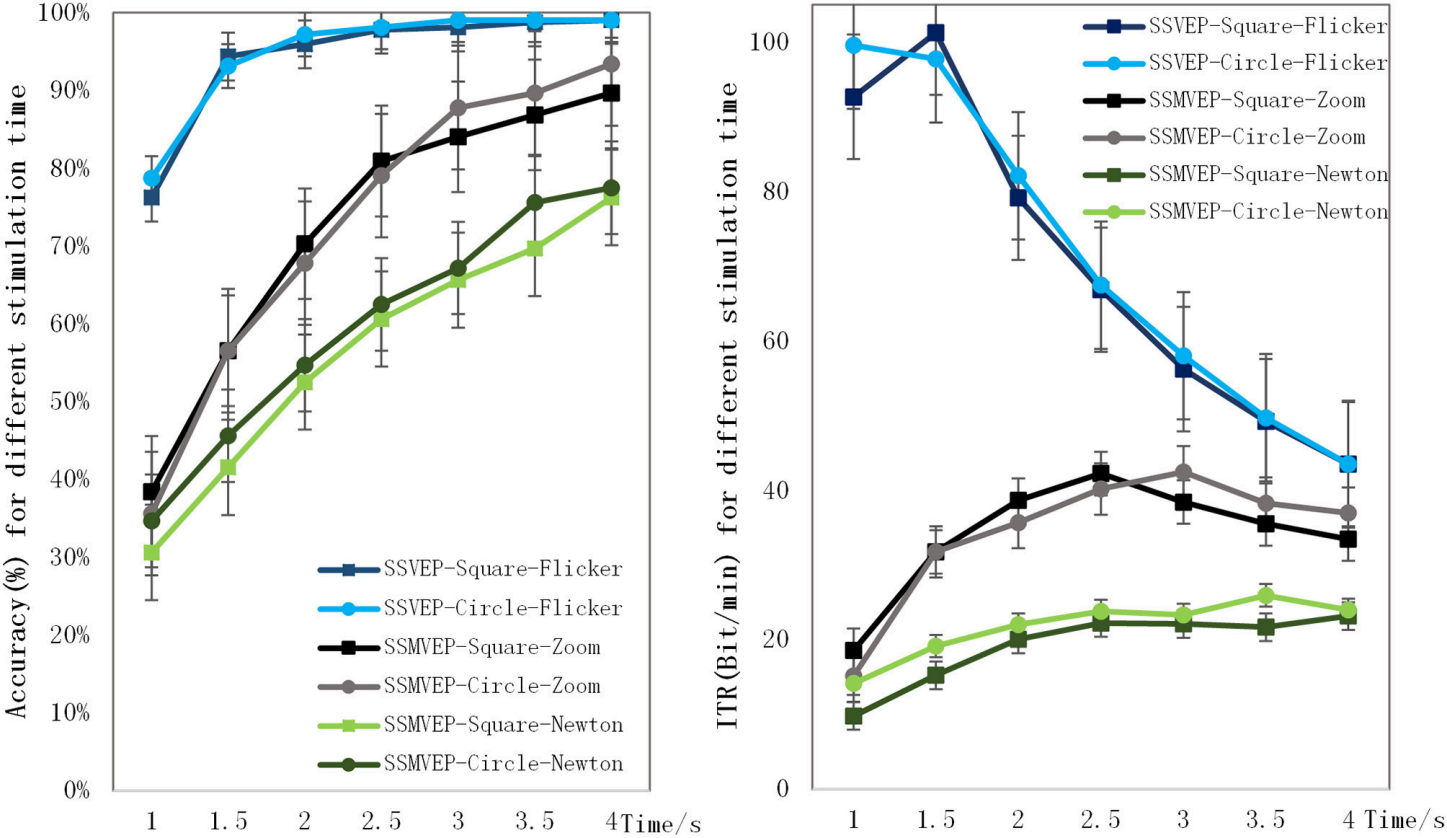
Recognition accuracy and ITR for different stimulation durations.

As shown in Figure 6, for the Flicker-based SSVEP paradigm, the recognition accuracy is basically stable and a maximum after 2.5 s, and the ITR is maximized at 1.5 s. The SSMVEP based on radial motion increased and reached a maximum in 4 s, and the ITR of the circular radial motion stimulation paradigm reached a maximum of 42.5 bit/min in 3 s for the square radial motion. The ITR of the stimulus paradigm reached a maximum of 42.2 bit/min within 2.5 s. The SSMVEP stimulation paradigm based on Newton's ring motion also reached a maximum at 4 s, and its ITR reached a maximum of 26.0 bit/min at 3.5 s.

### Visual Comfort Evaluation

**Figure 7:**
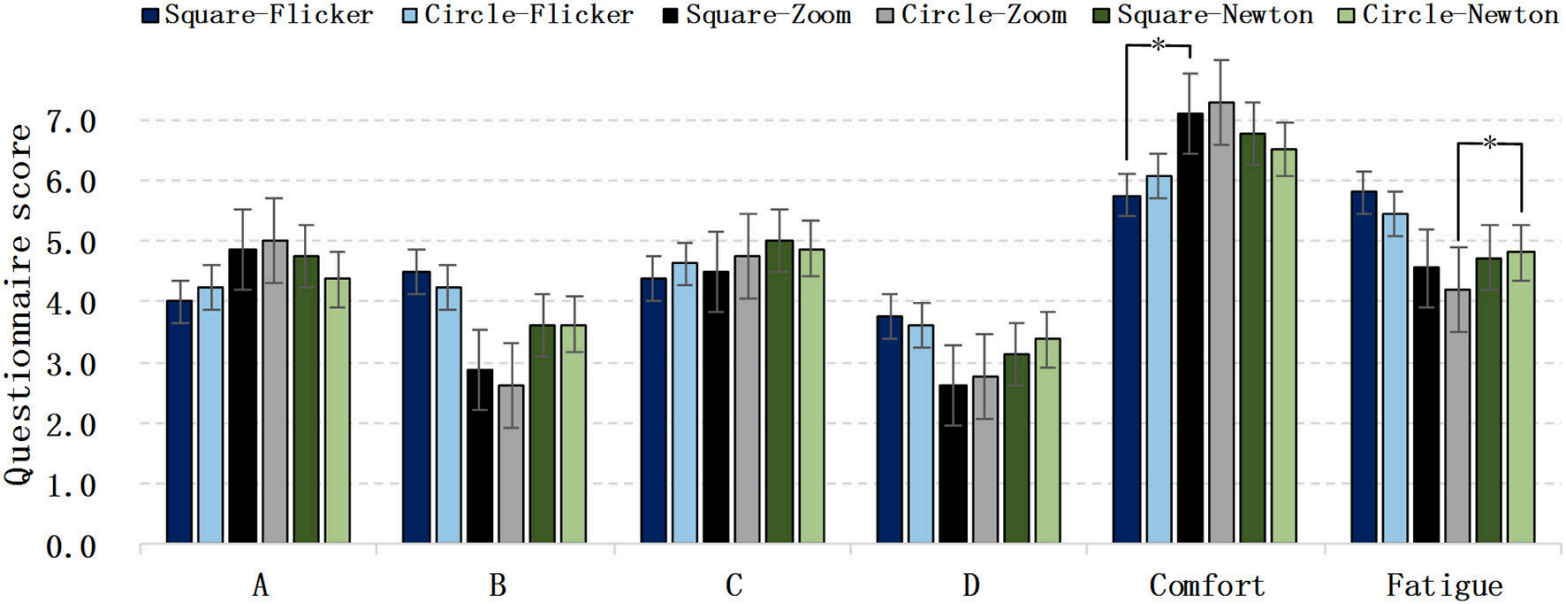
Visual comfort evaluation of different paradigms.

Figure 7 shows the score for each comfort question. There was no significant differences in the comfort scores of the square and circle flicker SSVEP paradigms. The comfort score of the SSMVEP based on radial zoom motion was significantly greater than that of the flicker-based SSVEP paradigm, and slightly greater than the SSMVEP paradigm based on Newton's ring motion, for which the average comfort score of the circular radial zoom motion was the greatest.

**Figure 8:**
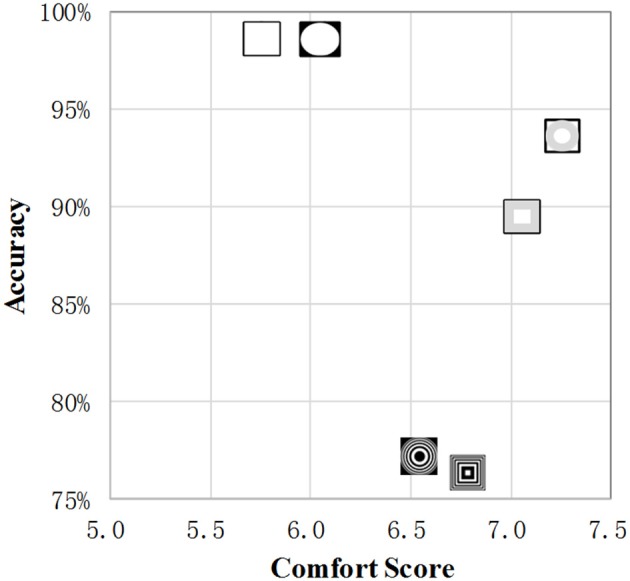
The comfort score and recognition accuracy.

In Figure 8, the horizontal and vertical axes represent the average comfort score and recognition accuracy of the various paradigms, respectively. Although the average recognition accuracy of the flicker-based SSVEP is close to 99%, the comfort score was lower than those of the four SSMVEP paradigms. The accuracy of the square and circular radial zoom motions were greater for the SSVEP paradigm, which was close to 90%, and its recognition accuracy was greater than that of the SSMVEP based on the Newton's and square ring motions.

### Recognition Accuracy Reduced by Fatigue

**Figure 9:**
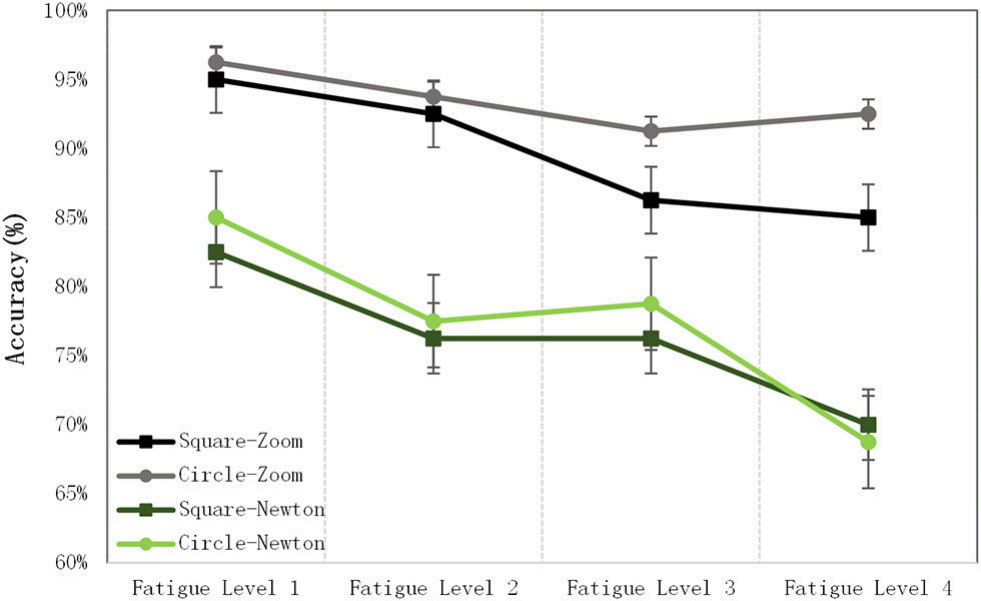
Recognition accuracy for different fatigue levels.

The recognition accuracies under different fatigue levels are shown in Figure 9. The recognition accuracies of all of the SSMVEP paradigms were reduced from fatigue levels 1 to 4. The recognition accuracy of the SSMVEP based on Newton's ring motion was reduced by 16%, while the recognition accuracy of SSMVEP paradigm based on circular radial zoom motion only decreased by 4%.

**Figure 10:**
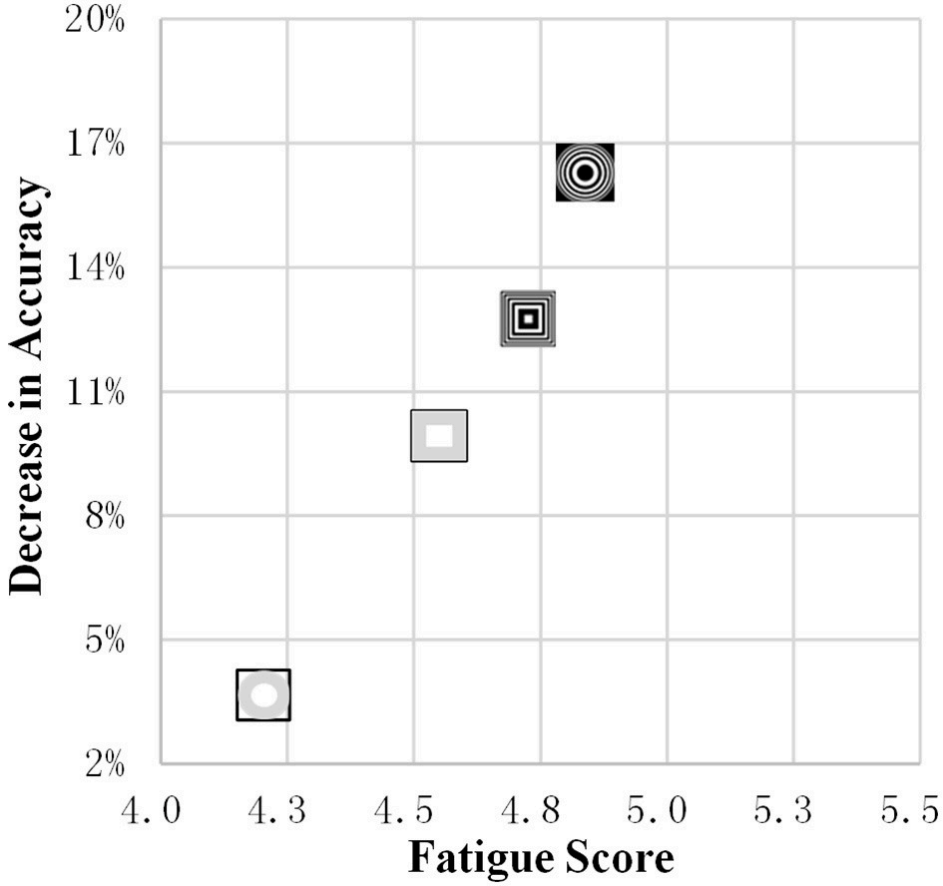
The fatigue score and decrease in accuracy.

In Figure 10, the horizontal and vertical axes represent the fatigue score of each SSMVEP paradigm, and the difference in the recognition accuracy between fatigue levels 1 and 4, respectively. A paradigm with a high fatigue score is associated with a greater decrease in the accuracy. The SSMVEP paradigm based on circular radial zoom motion had the lowest fatigue score and the lowest decrease in accuracy.

## Discussion

The zoom motion paradigm at a certain frequency can induce an SSMVEP. There are two parallel visual pathways in the visual system. Compared with the traditional stimulus generated by the brightness change of the graphic, the size of the sinusoidal modulation is used to generate the zoom motion, which not only activates the “what” path that perceives shape and color, but also activates the “where” path that perceives motion (Heinrich and Bach, 2003; Müller-Putz et al., 2005). Due to the depth perception of the human eye (Chang et al., 2014), the zoom motion paradigm causes a space movement sensation. Although the spectral response of the SSMVEP based on the radial zoom motion was not significantly greater than that of the SSVEP based on flicker, utilizing the CCA-based target frequency recognition algorithm for the sinusoidal motion paradigm could be used to achieve a higher accuracy and ITR.

Compared with the SSMVEP paradigm based on Newton's ring motion, the SSMVEP based on zoom motion had no obvious differences. In the experiment, one subject reported that the Newton's ring motion caused a feeling of vertigo, and provided a very low recognition accuracy of the square ring motion. Even if the sample was excluded, the average recognition accuracy of Newton's ring motion for the eight targets was still significantly lower than that of the recognition accuracy of the four targets in a previous study (Kremláek et al., 2004), the SSMVEP paradigm based on Newton's ring motion may not be suitable for a multiple target BCI system. The low recognition accuracy of Newton's ring motion-based SSMVEP may be related to its complicated graphic and motion patterns. In this paper, the target recognition accuracy of the SSMVEP based on the circular radial zoom motion reached 93.4%, which was greater than the recognition accuracy of the radial contraction-expansion motion paradigm in a previous study (Chang et al., 2014). This may be due to the complexity of the stimulus graphics, whether the SSMVEP induced by the same motion pattern of different graphics were different; this requires further research.

From the subjective comfort evaluation, the differences in the accuracy, and comfort between different shapes of flicker-based SSVEPs were insignificant, which is consistent with previous studies (Duszyk et al., 2014). However, the comfort of the flicker paradigm was significantly lower than that of the SSMVEP-based paradigm, while the visual comfort based on the Newton's ring motion paradigm was lower than that of the radial zoom motion paradigm. The SSMVEP target recognition accuracy based on circular radial zoom motion was significantly higher than that of the SSMVEP paradigm based on square radial zoom motion. The SSMVEP paradigm based on radial zoom motion was as editable as the traditional flicker-based SSVEP paradigm. The visual comfort and recognition accuracy of the SSMVEP paradigm can be improved by changing the stimulus color, shape, and size.

Regardless of the chosen SSVEP or SSMVEP-based BCI system, the performance and accuracy of the recognition was affected by increasing the duration. Therefore, the development of a visual stimulation paradigm with a lower accuracy reduction due to visual fatigue is required (Cao et al., 2014). The SSMVEP paradigm based on radial zoom motion proposed in this paper is not only more comfortable than the traditional flicker-based SSVEP paradigm, but also its recognition accuracy is greater than the Newton's ring SSMVEP paradigm in the previous study. Moreover, its accuracy decrease caused by fatigue was also less. The SSMVEP stimulation paradigm for a longer duration should be tested, and the objective fatigue evaluation will be addressed in a future study. Obtaining comparisons with other various SSMVEP paradigm, the potential advantages of the novel SSMVEP paradigm in fatigue-induced performance degradation problems would be proved.

## Ethics Statement

This study was carried out in accordance with the recommendations of Beihang University Ethics Committee with written informed consent from all subjects. All subjects gave written informed consent in accordance with the Declaration of Helsinki. The protocol was approved by Beihang University Ethics Committee.

## Author Contributions

XC and HN conceived and designed the study. XC, ZZ, GL, and KG performed the experiments. XC wrote the paper. ZZ, KG, and HN reviewed and edited the manuscript. All authors read and approved the manuscript.

### Conflict of Interest Statement

The authors declare that the research was conducted in the absence of any commercial or financial relationships that could be construed as a potential conflict of interest.
